# Oxidative Stress Regulation on Endothelial Cells by Hydrophilic Astaxanthin Complex: Chemical, Biological, and Molecular Antioxidant Activity Evaluation

**DOI:** 10.1155/2017/8073798

**Published:** 2017-09-27

**Authors:** M. Zuluaga, A. Barzegari, D. Letourneur, V. Gueguen, G. Pavon-Djavid

**Affiliations:** ^1^INSERM U1148, Laboratory for Vascular Translational Science, Cardiovascular Bioengineering, Paris 13 University, Sorbonne Paris Cité 99, Av. Jean-Baptiste Clément, 93430 Villetaneuse, France; ^2^Research Center for Pharmaceutical Nanotechnology, Tabriz University of Medical Sciences, Daneshgah Street, Tabriz 51656 65811, Iran

## Abstract

An imbalance in the reactive oxygen species (ROS) homeostasis is involved in the pathogenesis of oxidative stress-related diseases. Astaxanthin, a xanthophyll carotenoid with high antioxidant capacities, has been shown to prevent the first stages of oxidative stress. Here, we evaluate the antioxidant capacities of astaxanthin included within hydroxypropyl-beta-cyclodextrin (CD-A) to directly and indirectly reduce the induced ROS production. First, chemical methods were used to corroborate the preservation of astaxanthin antioxidant abilities after inclusion. Next, antioxidant scavenging properties of CD-A to inhibit the cellular and mitochondrial ROS by reducing the disturbance in the redox state of the cell and the infiltration of lipid peroxidation radicals were evaluated. Finally, the activation of endogenous antioxidant PTEN/AKT, Nrf2/HO-1, and NQOI gene and protein expression supported the protective effect of CD-A complex on human endothelial cells under stress conditions. Moreover, a nontoxic effect on HUVEC was registered after CD-A complex supplementation. The results reported here illustrate the need to continue exploring the interesting properties of this hydrophilic antioxidant complex to assist endogenous systems to counteract the ROS impact on the induction of cellular oxidative stress state.

## 1. Introduction

While reactive oxygen-derived species are the product of normal aerobic metabolism, they can also be produced at elevated rates under pathophysiological conditions [1]. As a consequence an alteration in the redox signaling leads to uncontrolled reactions between free radicals and neighboring molecules such as proteins, lipids, nucleic acids, and carbohydrates, inducing an imbalance in the redox homeostasis [2, 3] and thus originating a range of abnormalities further associated with chronic diseases. The apparition of abnormalities associated with vascular diseases was shown to be related with ROS production in the vessel wall [4]. Carotenoids like antioxidants have been investigated due to their capacity to moderate the damaging effects of ROS [5]. According to Britton [6], to be an effective antioxidant, carotenoids must react with free radicals in order to inhibit formation of harmful products by disrupting free radical chain reactions. Additionally, carotenoids can serve as a lipid peroxyl radical quenching either by the addition or abstraction of a hydrogen atom, or by electron transfer [7]. Tapiero et al. [8] attributed the singlet molecular oxygen and peroxyl radical scavenger action against photooxidative process to carotenoids.

An important aspect to bear in mind is that some carotenoids can switch from antioxidants to prooxidants. Among the factors that may trigger such change are the excessive increase of carotenoid concentration, high partial pressure of oxygen and oxidative stress which speeds up ROS production, and the capacity of carotenoids to interact and localize within membranes [9]. Besides the toxic effect carried out by high carotenoid concentration, controlled amounts may lead to the activation of signaling pathways able to recognize potential threats [10], particularly the Nrf2 transcription factor, in which antioxidants are believed to exert an indirect action [11].

**Figure 1:**
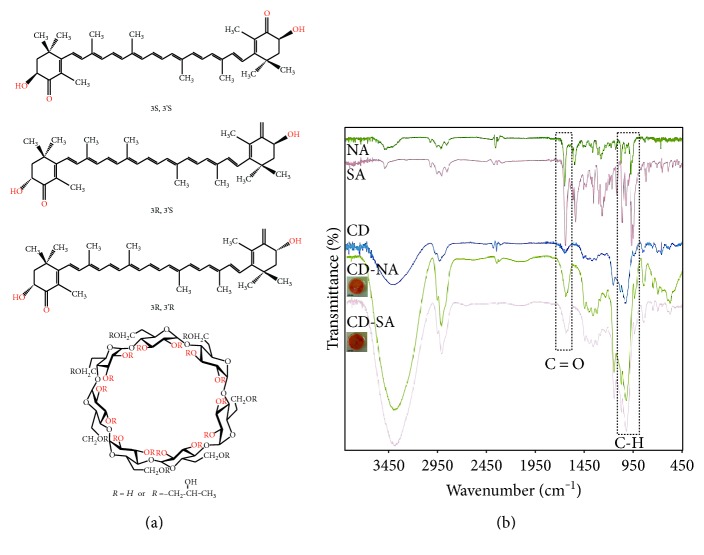
CD-A complex characterization. (a) Chemical structure of astaxanthin: 3S, 3′S; 3R, 3′S; 3R, 3′R esterification and hydroxypropyl-*β*-cyclodextrin (CD), respectively. (b) FT-IR spectrums of NA, SA, CD, CD-NA, and CD-SA.

Astaxanthin, a xanthophyll carotenoid, shows interesting and strong antioxidant and anti-inflammatory properties [12, 13]. Among the available sources of astaxanthin, two are of relevant importance: the natural (from microalgae *Haematococcus pluvialis*) and the produced via chemical synthesis. In the present work, both sources of astaxanthin in free form (without esterification) and purified by high-performance liquid chromatography, containing different stereoisomers, were used. Synthetic astaxanthin presents the isomers 3R, 3′R′, 3S, 3′S, and 3R, 3′S while 3S, 3′S is the only stereoisomer present in the natural source, Figure 1(a) [14–16]. Different antioxidant activities of both astaxanthin have been reviewed [17].

Owing to its structure, astaxanthin acts not only as a chain-breaking scavenger of free radicals but also as an inhibitor of lipid peroxidation [18]. In contrast to beta-carotene, the polar characteristics of astaxanthin allow it to preserve the membrane structure showing a significant antioxidant activity while avoiding a prooxidant effect [19, 20]. Additionally, the indirect antioxidant capacity of astaxanthin was also shown to potentially contribute to the regulation of gene expression [21–23]. As a highly unsaturated molecule, astaxanthin has a low water solubility and can be easily degraded by light, oxygen, and temperature, leading to the decrease of its bioavailability and a diminution of its properties [24]. Coombes et al. reported the natural astaxanthin plasma absorption up to 0.19 *μ*mol/L into the blood after 1 to 12 mg human intake for 1 year. The authors did not found significant effect on the oxidative stress reduction after oral ingestion in renal transplant recipients and suggested that the lack of effect could be due to the lower doses or to the length of the treatment [25].

At exposed by Forman et al., low antioxidant bioavailability could be convenient in order to prevent these molecules from acting as prooxidants; however, encapsulation systems, besides being a limitation to this regard, protect the molecule externally before ingestion to avoid further degradations [10]. Among the encapsulation strategies attempted for astaxanthin protection [26–29], the molecular inclusion with cyclodextrins has shown interesting results regarding astaxanthin solubility and stability [30, 31]. This research focused on the direct and indirect evaluation of the protective effect of hydroxypropyl-*β*-cyclodextrin-astaxanthin (CD-A) complex on human endothelial cells under exogenous oxidative stress. The synthesis and characterization of CD-A complex was successfully achieved. To verify the correct encapsulation of astaxanthin within the system and the sensitivity of this new complex to oxidation, direct, CD-A radical scavenging was quantified using AAPH, ABTS^•+^, and DPPH^•^ assays after *in tube* generation of peroxyl (ROO•), and alkoxyl (RO•) radicals. Additionally, *in vitro* tests allow the evaluation of CD-A complex interaction with target molecules such as protein side chain, unsaturated fatty acid, and other reactive oxygen species after t-BuOOH stress induction [32, 33]. Superoxide radical production was generated after mitochondrial depolarization after induction by antimycin A, thus blocking the electron transfer [34], while lipid peroxyl radicals were initiated by addition of Cumene hydroperoxide (CumOOH) [35]. An indirect antioxidant capacity evaluation was performed by understanding the molecular mechanisms involved in the regulation of endothelial cell gene expression by CD-A complex.

## 2. Materials and Methods

### 2.1. Chemical Reagents

Synthetic astaxanthin (SA; purity > 97.3%, powder, Lot: 40816) was purchased from Dr. Ehrenstorfer Co. Ltd. (LGC Standards, Germany). Natural astaxanthin (NA; purity > 97% HPLC, powder, Lot: 5M4707V), hydroxypropyl-*β*-cyclodextrin (CD; DS = 0.67), MitoTEMPO (SML0737; >98% HPLC), N-acetyl-L-cysteine (NAC; A9165), and antimycin A (*Streptomyces* sp.) were purchased from Sigma-Aldrich Co. LLC (Saint Louis, MO, USA); as well as the antioxidant standard 6-hydroxy-2,5,7,8-tetramethylchroman-2-carboxylic acid (Trolox, Lot: BCBJ8170V), 2,2′-azino-bis(3-ethylbenzothiazoline-6-sulfonic acid) diammonium salt (ABTS, Lot: 061M538V), potassium persulfate (≥99%, Ref: 216224), Cumene hydroperoxide (≥99%, Ref: 247502), 3-(4,5-dimethyl-2-thiazolyl)-2,5-diphenyl-2H-tetrazolium bromide (MTT), *tert*-butyl hydroperoxide (*t*-BuOOH, Lot: BCBJ2885V), 2,2′-azobis(2-methylpropionamidine) dihydrochloride (AAPH, Ref. 440914), fluorescein (Ref. F6377), 2,2-diphenyl-1-picrylhydrazyl (DPPH, Ref: D9132), Power SYBR Green Master Mix (ABI Biosystem, Ref: 4472908), dimethyl sulfoxide (DMSO, Lot: SZBD1830V), and isopropanol (70% in H_2_O, Ref: 563935). 5-(and 6-)Chloromethyl-2′,7′-dichlorodihydrofluorescein diacetate, acetyl ester (CM-H_2_DCFDA, Lot: 1600227) and C11-BODIPY® 581/591 (Lipid Peroxidation Sensor, D3861) were purchased from Life Technologies (Invitrogen, Eugene, OR, USA). MitoSOX Red was purchased from Thermo Fisher Scientific (Oregon, USA). Acetone (HPLC gradient grade), methanol (HPLC gradient grade), and chloroform (HPLC gradient grade) were purchased form Carlo Erba Reagents S.A.S (France). Cell culture reagents were all purchased from Gibco (Life Technologies, Carlsbad, CA, USA). Double distilled and deionized water was used for all the experimentation process.

### 2.2. Preparation of CD-A Complex

Natural and synthetic astaxanthin (NA and SA) were included into CD according to the method presented by previous authors [30, 36], with minor modifications. Briefly, 1 mL of NA or SA in acetone/chloroform (*v/v* 1 : 1) solution (1 mg/mL) was added to 250 mg of CD dissolved in 12.5 mL of 95% methanol (20 mg/mL) in a 25 mL flask filled with nitrogen. The mixture was sonicated (5 min Ultrasonic bath BANDELIN SONOREX RX-100-H) and stirred for 24 h at 35°C in a dark chamber. Subsequently, the solution was subjected to a vacuum concentrator and recovered with distilled water. The final solution was frozen, lyophilized (Cryotec, Lyophilizer Crios, Saint Gely du Fesc, France), and stored at −4°C.

### 2.3. Characterization of CD-A Complex

To establish the astaxanthin content into the complexes, a calibration curve of SA (5 mg/10 mL DMSO) absorbance at 480 nm was plotted against concentrations (0–20 *μ*M; UV/VIS spectrophotometric measurements, Perkin Elmer Lambda 12 spectrophotometer). Once the curve was established, the complexes (5 mg/1 mL DMSO) were analyzed by UV/VIS spectroscopy and the concentration and inclusion rate of astaxanthin into the complex were calculated according to Chen et al. [30] as seen in 1. Where *C*_astaxanthin_ is the astaxanthin content in the inclusion complex (g/mL); *W_i_* is the total weight (g) of inclusion complex; *V*_*c*_ is the volume (mL) of the solvent used for quantification analysis; *W*_*a*_ is the weight of astaxanthin (g) used for inclusion complex preparation; and *W*_*c*_ is the weight of complex (g) used for quantification.

(1) $$\begin{array}{c} Inclusion\;rate\;\left(\%\right)=\frac{{C_{astaxanthin}}^{*}{W_{i}}^{*}V_{c}}{{\left({W_{a}}^{*}W_{c}\right)}^{*}100}. \end{array}$$

FT-IR characterization corroborates the astaxanthin inclusion within CD. A homogenized powder was obtained by mixing a sample of CD-NA or CD-SA with 200 mg of KBr. Then, each mix powder was placed into a sampling cup, smoothed, and compressed into the holder using a compression gauge. The obtained compact mixture was placed into the IR spectrometer (AVATAR 370, Thermo Spectra Tech Inc., Shelton, CT, USA), and spectral curves in the ranges of 400–4000 cm^−1^ were recorded using OMNIC Software (Thermo Fisher Scientific Inc., Waltham, MA, USA).

### 2.4. Direct CD-A Antioxidant Activity Measurement

#### 2.4.1. Trolox Equivalent Antioxidant Capacity (TEAC) Assay

Antioxidant capacities of CD-NA and CD-SA complexes were measured using the TEAC assay protocol presented by Re et al. [37] with slight modifications. Here, the ABTS radical cation ABTS^•+^ is obtained by the oxidation of ABTS (7 mM) with potassium persulfate (K_2_S_2_O_8_, 2.45 mM) in distilled water (vol/vol reaction). The resulting solution was placed in the dark at room temperature for 12–16 h before use. CD-NA, CD-SA (0–5 *μ*M, 50 *μ*L), or Trolox standards (0–200 *μ*M, 50 *μ*L) were mixed with 1 mL of ABTS^•+^ (absorbance maximum of 0.70 ± 0.02 at 734 nm). The absorbance of the mixture solution was recorded after 1 h of incubation at 30°C (734 nm, UV/VIS Lambda 12, PerkinElmer Inc., Norwalk, CT, USA). Inhibition percentage was calculated using 2. The Trolox equivalent antioxidant capacity (TEAC) was defined as the millimolar concentration of Trolox with the same antioxidant activity as 1 mM concentration of the samples [38].

(2) $$\begin{array}{c} Scavenging\;rate\;\left(\%\right)=\frac{{\left[{ABTS}^{+}\right]}_{initial}-{\left[{ABTS}^{+}\right]}_{final}}{{\left[{ABTS}^{+}\right]}_{initial}}*100. \end{array}$$

#### 2.4.2. The Oxygen Radical Antioxidant Capacity (ORAC) Assay

The oxygen radical antioxidant capacity of CD-NA and CD-SA complexes was evaluated [32, 39]. Fluorescein solution (4 nM, 150 *μ*L) was mixed with CD-NA (0–5 *μ*M, 25 *μ*L), CD-SA (0–5 *μ*M, 25 *μ*L), Trolox standards (1–18 *μ*M, 25 *μ*L) or blanks, and AAPH (160 mM, 25 *μ*L) in a 96-well microplate. The plate was incubated at 37°C, and fluorescence decay was monitored during 1 h with data taken every minute using an emission/excitation filter of 485–528 nm (i-control™ microplate reader software, TECAN Männedorf, Switzerland). The complex antioxidant activity was determined from its ability to keep the fluorescence signal of the indicator in the presence of peroxyl radicals. The AUC and AUC_net_ were calculated using 3 and 4. Where *f*_1_ is the initial fluorescence reading at 0 min and *f*_i_ is the fluorescence measured at time *i*. Final ORAC values were calculated by using the regression equation between the Trolox concentration and the AUC_net_ and were expressed as Trolox equivalents (TEAC) as millimolar per liter.

(3) $$\begin{array}{c} AUC=0.5+\left(\frac{f_{2}}{f_{1}}\right)+\left(\frac{f_{3}}{f_{1}}\right)+\cdots +0.5\left(\frac{f_{n}}{f_{1}}\right). \end{array}$$

(4) $$\begin{array}{c} {AUC}_{net}={AUC}_{sample}-{AUC}_{blank}. \end{array}$$

#### 2.4.3. CD-A Antioxidant Activity by DPPH

CD-A complex antioxidant activity was evaluated using the DPPH-free radical assay [40]. Briefly, DPPH (0.16 mM; 1.5 mL) was added to 2 mL of different sample CD-A complexes (0–5 *μ*M), Trolox standards, or blank in ethanol solution. The mixture was incubated for 30 min in the dark at 25°C. Antioxidant effect was evaluated by following the decrease of UV absorption at 517 nm. The radical scavenging activity was calculated using 5. Where *A*_*c*_ is the DPPH solution absorption, *A*_*i*_ is the DPPH solution absorption after sample addition, and *A*_*j*_ is the initial absorption of the experimental samples.

(5) $$\begin{array}{c} Scavenging\;rate\;\left(\%\right)=\frac{A_{c}-\left(A_{i}-A_{j}\right)}{A_{c}}*100. \end{array}$$

### 2.5. Cell Culture

Human umbilical vein endothelial cells (HUVECs) were purchased from ATCC (CRL 1730). Cells were grown in minimum essential medium-L-glutamine (MEM), supplemented with 10% (*v/v*) fetal calf serum (FBS) and 1% penicillin-streptomycin-amphotericin (PSA). Cells were seeded in a T75 cell culture flask (Corning) and kept in a humidified incubator containing 5% CO_2_ at 37°C. The culture medium was replaced twice every week, and the cells were split 1 : 3 every week.

### 2.6. Evaluation of CD-A Cytotoxicity in HUVEC by MTT Reduction Assay

Cell viability was assessed using MTT assay. Briefly, HUVEC density of 2.10^4^ cells/well was seeded and cultured overnight. Cells were treated with different samples: CD-NA and CD-SA (0–5 *μ*M), any antioxidant (culture medium MEM, negative toxicity control, NTC), or 10% DMSO (positive toxicity control, PTC) during 24 h or 48 h. Afterwards, all solutions were washed and cells were incubated during 3 h at 37°C with 200 *μ*L of MTT solution (0.5 mg/mL). Then, all wells were washed out of MTT solution and 200 *μ*L of isopropanol were added for 20 min to solubilize the formazan crystals. The optical density was recorded at 490 nm (i-control microplate reader software, TECAN Männedorf, Switzerland). Not cytotoxicity of samples was considered if cellular viability was >70% of the control (based on the ISO 10993 : 2009 regarding the biological evaluation of medical devises).

### 2.7. In Vitro Inhibition of Cellular Reactive Oxygen Species and Lipid Peroxidation by CD-A Complex

The capacity of CD-NA and CD-SA complexes to reduce the cellular levels of ROS and lipid peroxides in human endothelial cells was determined using the fluorescence probes CM-H_2_DCFDA, MitoSOX Red, and C11-BODIPY, respectively. Prior to tests, HUVECs were removed from growth media and detached with trypsin after reached the 80% of confluence. A density of 1.10^4^ cells/well was seeded in a 96-well cell culture plates and incubated overnight at 37°C with 5% CO_2_. Low glucose-MEM was used to prepare antioxidant samples: CD-NA and CD-SA samples (0–5 *μ*M), NAC (500 *μ*M), and MitoTEMPO (500 nM). Hank's buffered salt solution (HBSS) was used to solubilize the probes.

#### 2.7.1. Cellular ROS Measurement

Cellular antioxidant activity of CD-NA and CD-SA complexes was quantified using the cell permeable probe CM-H_2_DCFDA. This probe emits no fluorescence in its original state. After crossing the cell membrane, DCFH-DA is hydrolyzed by intracellular esterases into DCFH, which becomes fluorescent once oxidized to DCF in the presence of ROS. The accumulation of DCF inside the cells is measured by the fluorescence increase at 530 nm using an excitation wavelength of 485 nm in a kinetic mode [41]. HUVECs were incubated overnight with medium containing CD-NA (5 *μ*M), CD-SA (5 *μ*M), or reference antioxidants NAC (500 *μ*M), and MitoTEMPO (0.5 *μ*M). After 24 h, the cells were washed twice with PBS to remove the medium and CM-H_2_DCFDA was added to the wells (5 *μ*M final concentration) during 1 h, under light protection. Oxidative stress was induced by the subsequent addition of t-BuOOH (100 *μ*M) or antimycin A (200 *μ*M, during 20 min), excluding the blank wells. Variations in the fluorescence intensity were recorded during 60 min with data taken every 5 min (i-control microplate reader software, TECAN Männedorf, Switzerland). The capacity of CD-A to reduce the oxidative cell environment was evaluated as expressed in (6), where *I*_*cn*_ and *I*_*sn*_ represent the intensity of cells exposed to t-BuOOH or antimycin A without and with antioxidant presence at time *n*, respectively.

(6) $$\begin{array}{c} CAA\left(\%\right)=\frac{I_{cn}-I_{sn}}{I_{cn}}*100. \end{array}$$

#### 2.7.2. Mitochondrial ROS Detection

The capacity of CD-NA and CD-SA complexes to inactivate mitochondrial superoxide production was measured using MitoSOX Red fluorescent probe. MitoSOX Red after being target to the mitochondria, it is oxidized by superoxide to form 2-hydroxymitoethidium [42]. HUVECs were incubated overnight with medium containing CD-NA (5 *μ*M), CD-SA (5 *μ*M), or reference antioxidants NAC (500 *μ*M), and MitoTEMPO (0.5 *μ*M). After 24 h, the cells were washed twice with PBS to remove the medium and incubated with MitoSOX (5 *μ*M), for 20 minutes at 37°C, protected from light. Cells were gently washed three times with warm buffer. Reactive oxygen species were induced by the addition of antimycin A (AA; 200 *μ*M, during 20 min), excluding the blank wells. Fluoresce was monitored at *λ*ex/*λ*em = 510/595 nm (single-read measure; i-control microplate reader software, TECAN Männedorf, Switzerland). CD-A effect in decreasing mitochondrial ROS generation was compared to the percentage of control (not stressor or antioxidant treatment).

### 2.8. Cellular Lipid Peroxidation Analysis

C11-BODIPY581/591 is a fluorescent fatty acid analogue which allows the quantification of lipid peroxidation by indirect measure of mitochondrial ROS production [43]. Upon free radical-induced oxidation, its fluorescent properties shift from red to green [44]. Ninety-six well plates containing the cells were washed with PBS, before addition of C11-BODIPY (5 *μ*M, during 30 min). Cells were incubated with 5 *μ*M of CD-NA and CD-SA for 1 h, under light protection. Lipid peroxidation was initiated by addition of Cumene hydroperoxide (CumOOH, 50 *μ*M). Fluoresce was monitored at red *λ*ex/*λ*em = 590/7, 632/45 nm and green *λ*ex/*λ*em = 485/14, 520/10 nm (i-control microplate reader software, TECAN Männedorf, Switzerland). Percent of CD-A cellular lipid peroxide activity inhibition was calculated relative to the positive control fluorescence intensity (PC; CumOOH without antioxidant).

### 2.9. Indirect CD-A Antioxidant Activity Measurement

#### 2.9.1. mRNA Extraction and Real-Time RT-PCR

HUVECs were seeded in 6-well plates. Then, CD-A (8 *μ*M) was added to the cells and incubated for 24 h. After discarding the supernatant, the cells were washed twice with PBS and incubated with AAPH (5 mM) or t-BuOOH (25 *μ*M) for 24 h (the IC50 of AAPH and or t-BuOOH at 24 h were determined 5 mM and 25 *μ*M, resp., (data not showed)). Total RNA was extracted using the TRIzol® reagent (Invitrogen TM, Life Technologies). The RNA yield and purity were determined using a NanoDrop ND-1000 spectrophotometer. For RT-PCR, 1 *μ*g of RNA, 0.4 *μ*m universal hexamer primer, 1 *μ*L dNTP (10 Mm), and DEPC water mixed and incubated at 65°C for 5 min and kept on ice. Then, 5 U reverse transcriptase enzyme (MMLV), 1x RT buffer, and 1 U/*μ*L RNase inhibitor were added to reaction and the solution was increased to a volume of 20 *μ*L with DEPC water. Reverse transcription of mRNAs was performed at 25°C for 10 min and 42°C for 60 min. Then, real-time PCR was performed to measure expression levels of target genes (Table 1) using a power SYBR Green Master Mix (ABI Biosystem) on a Bio-Rad IQ5 real-time PCR detection system.

#### 2.9.2. Immunoblotting

Proteins were extracted from the treated HUVEC using radioimmunoprecipitation assay buffer (RIPA, 50 mM Tris-base, 1.0 mM EDTA, 150 mM NaCl, 0.1% SDS, 1% Triton X-100, 1% sodium deoxycholate, and 1 mM phenylmethylsulfonyl fluoride). Extracts were separated by 12% SDS-PAGE gel and transferred to a polyvinylidene difluoride membrane (Millipore, MA, USA) previously probed with primary antibodies (Abcam) specific to NQO1 (ab80588), PTEN (ab137337), HO-1 (ab52947), Nrf2, and GAPDH (ab37168) before being incubated with horse radish peroxidase-conjugated secondary antibody (1 : 2000, Sigma-Aldrich). Bands were detected using a chemiluminescent kit (ECL, Thermo, 32106). Experiments were performed in triplicate.

### 2.10. Statistical Analysis

All experiments were repeated at least three times to ensure the reproducibility of each test. The results were expressed as the mean ± SDE, and statistical analysis was done using one-way ANOVA followed by Tukey's HSD post hoc test (JMP Software, Version 9; SAS Institute, Cary, NC, USA). The results were considered significantly different if *p* value < 0.05.

For real-time PCR, the level of expression was calculated based upon the PCR cycle number (*C_T_*). The endogenous control GAPDH and RNU6 were used for normalization of mRNA and microRNA expression levels, respectively. *C_T_* values were used to calculate relative expression using SPSS Version 14.0 software by difference in the *C_T_* values of the target RNAs after normalization to RNA input level. Relative quantification was represented by standard 2^ΔCT^ calculations. Δ*C_T_* = (*C*_*T*-target gene_−*C*_*T*-GAPDH_). Each reaction was performed in triplicate.

## 3. Results and Discussion

### 3.1. Characterization of CD-A Complex

Here, both natural and synthetic astaxanthin were successfully included into the CD hydrophobic cavity (Figure 1, SD available online at https://doi.org/10.1155/2017/8073798), showing that stereoisomers did not constrain the inclusion process, as confirmed by FTIR measurements.  Figure 1(b) presents the representative absorption bands of astaxanthin at 1656–1652 cm^−1^, and 974–970 cm^−1^, which indicate the C = O and C-H stretching vibrations respectively; after CD inclusion, these bands were weaker, as reported by Qiu et al. [45].

According to the regression model suggested by Dong et al. [36], an inclusion rate of 12.05 ± 0.96% for CD-NA and 7.21 ± 0.64% in the case of CD-SA was obtained, with a weight recovery rate around 89% (Figure S1A-B). CD-NA and CD-SA concentrations after inclusion were 3.4 ± 0.4 *μ*M and 2.6 ± 0.4 *μ*M, respectively, calculated from the SA calibration curve and the CD-A OD curves at 480 nm (Figure S1C-D). No astaxanthin precipitation was noticed even when solubilized with high amounts of CD-A complex in DI water, which is in accordance with similar works [31, 46–48].

### 3.2. CD-A Antioxidant Quantification

Oxidative stress is produced by the action of different ROS; hence, efficient methods able to quantify the influence of external substances like antioxidants in the prevention of radical's formation are needed. Available indirect probes provide valuable information on changes on the redox environment of the cell, but many of these methods are not specific and do not allow subcellular localization, and their response is affected by different chemical interactions [49]. Despite that, these methods represent a valuable tool to obtain an overview of the antioxidant ability of several molecules such as antioxidants.

**Figure 2:**
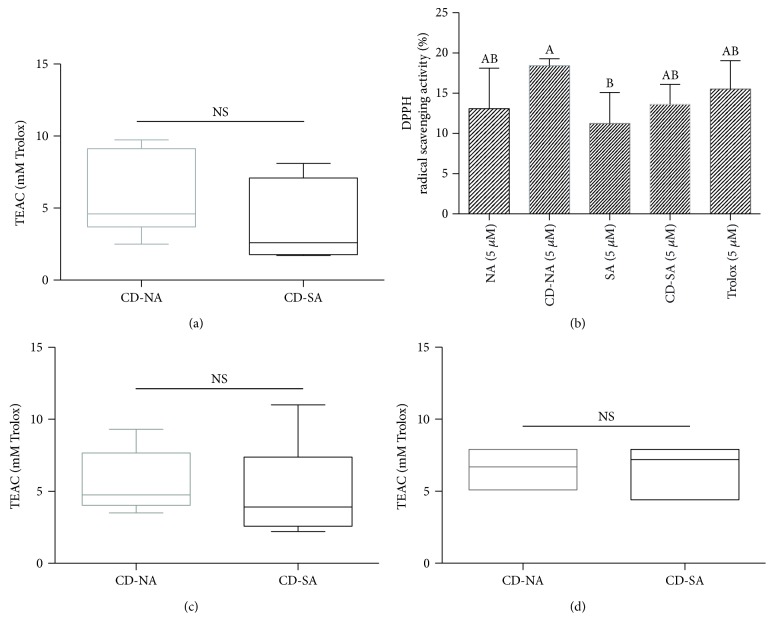
Chemical assays measuring the antioxidant capacity of CD-A complex. (a) TEAC CD-NA and CD-SA complexes measured by ABTS assay. (b) DPPH radical scavenging activity of free astaxanthin (NA and SA), CD-A complex, and Trolox antioxidant reference molecule. (c) Oxygen radical absorbance capacity (ORAC) assay of both complexes expressed as the Trolox equivalent in Mm. (d) CD-A complex antioxidant stability study after 6 months of storage at 6°C, under light protection, calculated by means of ORAC assay. Data are means ± SD of six experiments. Levels not connected by the same letter are significantly different (*p* < 0.05).

The antioxidant activity of CD-NA and CD-SA complexes was directly assessed by TEAC, DPPH, and ORAC assays. These chemical methods, while being simple, sensitive, and reproducible, provide useful information about the carotenoid antioxidant activity [50]. CD-NA and CD-SA scavenging capacities corresponded to 5.73 ± 2.9 and 3.93 ± 2.8 mM of Trolox, respectively, (Figure 2(a)), evaluated by the ABTS^•+^ assay. This result agreed with TEAC values reported by other authors for astaxanthin in its free form [39, 51–54]. ORAC assay was used to measure the capacity of CD-NA and CD-SA to inhibit the thermal decomposition of AAPH against alkyl (R•), peroxyl (ROO•), and alkoxyl (RO•) radicals, (where *R* = H2N(HN)C) [32, 39]. Here, ORAC values (5.73 ± 2.1 and 5.10 ± 3.10 mM of Trolox) were in the same range as those obtained by ABTS^•+^ assay (Figure 2(c)). It seems appropriate to refer to the scavenging capacities reported for esterified astaxanthin and synthetic astaxanthin, which according to the literature are in a range between 0.1 ± 0.25 and 2.43 ± 0.02 for natural and synthetic astaxanthin, respectively, using the ABTS assays and 1.68 ± 0.25 and 8.1 ± 1.12 using ORAC test [51, 53, 54]. The variability of these capacities could probably be due to the low miscibility of hydrophilic components with chemical products. Additionally, the preservation of CD-A antioxidant capacity was evaluated after 6 months of complex storage at 6°C under light protection.  Figure 2(d) presents an ORAC TEAC value in the order of 5 mM of Trolox for both CD-NA and CD-SA complexes, reflecting the successful conservation of astaxanthin in the CD cavity. Passed this time, a floc was observed in the vials, a behavior already described by Chen et al. [30], who observed a floc formation after 6 h of complex dispersion in water. However, the preservation of astaxanthin antioxidant capacity after 6 months revealed that even if astaxanthin precipitates, a new covalent bond could be induced by simply mixing the vial.

Regarding the DPPH radical quenching, an inhibition percentage around 18% was found for CD-A complex at astaxanthin concentration of 5 *μ*M, a not negligible value, since literature reported scavenging rate of 97% for a greater SA concentration (133 *μ*M) [55]. Here, we report a complex inhibition capacity against ROS directly reliant to the astaxanthin concentration within the complex. CD-NA and CD-SA complexes presented a similar antioxidant scavenging capacity as expressed by the TEAC value; however, their activity was stronger than the Trolox standard antioxidant molecule, evaluated by ABTS^•+^ and ORAC assays. No significant difference was observed between astaxanthin radical quenching before and after inclusion into the CD and Trolox, in the DPPH test. Some authors attribute the antioxidant capacity of astaxanthin, to the activation of the hydroxyl group by the keto group allowing the hydrogen transfer to the peroxyl radical, and thus acting as a chain breaking in the free radical reaction [56, 57]. The astaxanthin inclusion into the CD cavity took place due to a noncovalent link between the CD hydrophobic cavity and the hydrophobic molecule, enhancing the CD-drug interaction with the lipophilic environment [58], without affecting the antioxidant properties.

In this study, chemical probes were used in principle as a verification tool of the preservation of CD-A complex sensitivity to oxidation before the evaluation of biological antioxidant capabilities. However, authors want to recognize the variability and instability of these probes due to external factors like light, temperature, or pH, which can degrade the probe during the time of analysis. Despite the use of a reference antioxidant Trolox and expressing the results based on Trolox equivalents, obtained results using ORAC, TEAC, and DPPH assays may lead to different conclusions, agreeing with data reported for some antioxidants [59]. It is worthy to highlight that CD-A reacts differently with the reagents used in each test for the determination of its antioxidant capacity; moreover, its capabilities measured by these assays provide information about the chemical reactivity of these compounds to block ROS without referring to the *in vitro* or *in vivo* relevance on human health.

### 3.3. In Vitro Cytoprotective Activity of CD-A Complex

**Figure 3:**
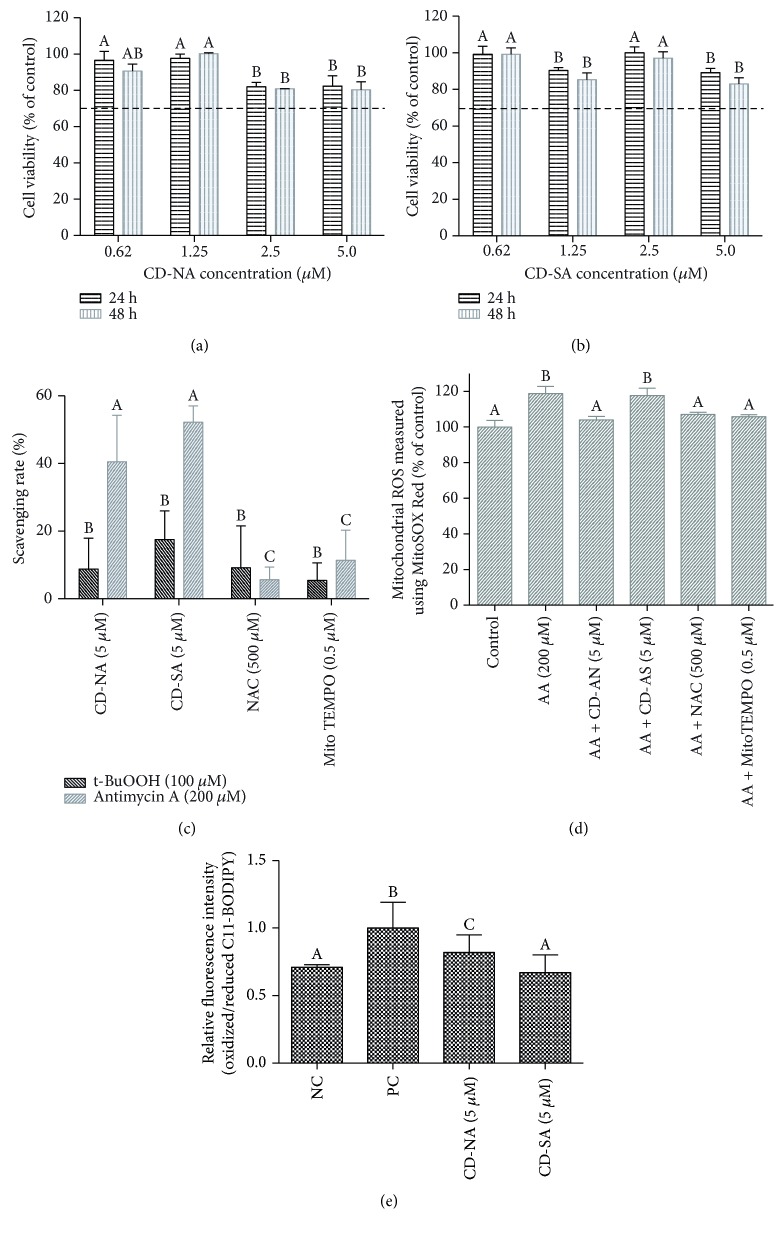
*In vitro* evaluation of CD-A complex cytocompatibility, CD-A complex protective effect against cellular ROS and lipid peroxidation. Cellular viability of HUVECs exposed to CD-A complex during (a) 24 h and (b) 48 h: doted lines represent a higher viability of 70% with regard to PTC (DMSO 10%), indicating a good cell CD-A complex compatibility. Cellular antioxidant activity (CAA) of CD-A complex measured by (c) CM-H_2_DCFDA and (d) MitoSOX Red: ROS were induced by either t-BuOOH or antimycin A in both assays. NAC and MitoTEMPO were used as antioxidant references. (d) Cellular lipid peroxidation activity (CLPAA) results for CD-A complex compared to positive control (HUVEC exposed to CumOOH). Data are means ± SD of six experiments. Levels not connected by the same letter are significantly different (*p* < 0.05).

CD have been currently used as a solubilizer for different kinds of hydrophobic molecules, and any up-to-date biocompatibility problem has been reported when exposed to diverse cell lines in concentrations not greater than 40 mg/mL [60–62]. Astaxanthin stability in culture medium is an important parameter conditioning cellular interaction and its antioxidant ability. Here, astaxanthin was included in CD complexes (CD-A) and thus stabilized in the culture medium at 37°C. As shown in Figures 3(a) and 3(b), CD-A complexes were noncytotoxic to HUVEC, represented by a cell viability exceeding 70% at concentrations up to 5 *μ*M after 24 and 48 h of exposure, showing a good biological acceptance of the complex by the HUVEC. A maximum decay of 20% of NTC was registered for the CD-SA at concentrations higher than 5 *μ*M, while CD-NA showed a faster decay at 2.5 *μ*M without exceeding 20% of NTC, indicating a suitable compatibility for both CD-A complex. These results agreed with the data reported for astaxanthin samples without inclusion, where a high cytoprotective potential was observed for concentrations lower than 25 *μ*M in a cell population of HUVEC, HepG2, and MCF-7 cells [51, 63]. Further, different CD concentrations were tested to verify their compatibility with HUVECs and no toxicity effects were remarked for concentrations lower than 40 mg/mL (data not shown).

### 3.4. Direct Biological Evaluation of CD-A Antioxidant Capability

In contrast to chemical assays which offer useful information of antioxidant activity of components, cellular tests take into account the bioavailability and metabolism of the tested compound providing information of ROS downstream effect in living cells [43]. Mechanisms of antioxidant/detoxifying protection are challenging. New approaches to detect ROS focused on the use of specific components to directly target organelles involve in ROS production. For instance, MitoSOX Red fluorescence probe is able to directly target complex III within the mitochondria to sense O_2_^•−^ production, to form 2-hydroxy-mito-ethidium [42]. Also, C11-BODIPY probe assessed the indirect measure of mitochondrial ROS production in living cells to extent lipid peroxidation [44]. Moreover, the CM-H_2_DCFDA assay provided information concerning general disturbance in the redox state of cells [49]. In this study, several methods and stressors were used to assess biological relevance and cellular availability of astaxanthin to block oxidative stress.

Additionally, the direct scavenger capacity of CD-A to neutralize the chemical-induced oxidative stress in HUVEC after either peroxyl (ROO•) or alkoxyl (RO•) radical generation using AAPH or t-BuOOH was evaluated. Additionally, superoxide radicals were induced by antimycin A, and lipid peroxyl radicals were initiated by addition of CumOOH. CD-A complex scavenging capacity was compared with two antioxidants that were shown to protect endothelial cells under induced oxidative stress [64, 65]: (a) NAC, a scavenger of free radicals such as hydroxyl radical, hydrogen peroxide, and superoxide [66], and (b) MitoTempo, a mitochondria-targeted superoxide dismutase-specific antioxidant.

Intracellular antioxidant capacity of CD-A evaluated by CM-H_2_DCFDA showed that HUVEC supplementation with either CD-NA or CD-SA during 24 h markedly inhibits superoxide radical induced by antimycin A. Scavenging rate of astaxanthin complexes (40–50%) was higher than that measured after incubation with NAC or MitoTEMPO (10–20%, *p* < 0.05; Figure 3(c)). Besides, RO• and ROO• radical reductions after t-BuOOH-induced stress were lower compared with CD-NA and CD-SA complexes (10–30%), NAC or MitoTEMPO (5–20%) treatments (nonsignificant differences). MitoSOX Red probe showed a significantly increased mitochondrial O_2_^•−^ levels in HUVEC under antimycin A ROS induction (Figure 3(d), *p* < 0.05). The redox equilibrium was reestablished after antioxidant supplementation (compared to nonstressed cells) showing similar levels for CD-NA, MitoTEMPO, and NAC (Figure 3(d)). Additionally, CD-A ability to protect HUVEC from lipid peroxidation was evaluated. Lipophilic C11-BODIPY probe was monitored as indicator of peroxidative membrane damages after addition of CumOOH, a lipid peroxide radical initiator [22]. As showed in Figure 3(e), a reduction in the cellular lipid peroxidation state was observed when HUVECs were supplemented with CD-NA and CD-SA complexes. The astaxanthin capacity to inhibit the penetration of oxidative substances across the lipid membrane by blocking the initiation of a lipid peroxidation process was confirmed by other authors [41, 64]. Here, we demonstrate the preservation of astaxanthin activity into the complexes.

Interestingly, intracellular antioxidant activities of CD-NA and CD-SA complexes are higher than those reported for astaxanthin stereoisomers and free form without inclusion [51, 67–69]. Moreover, we previously showed the CAA protective capacity exerted by algae natural astaxanthin in human endothelial cells subjected to oxidative stress *in vitro* [51]. Recently, Xue et al. [70] demonstrated that astaxanthin treatment markedly attenuated mitochondrial ROS produced after total body irradiation. Moreover, a potent mitochondrial ROS reduction had been noticed when using targeted than nontargeted mitochondrial antioxidants in different *in vitro* and *in vivo* models [64, 70–74]. In contrast to synthetic astaxanthin, natural astaxanthin without purification also contains other carotenoids, and there is no much evidence confirming the fact that these carotenoids act in synergy, in an additive manner or possibly cancel each other [17]. The results showed here did not confirm or reject the anterior idea but support the fact that similar antioxidant activities are obtained for either natural or synthetic astaxanthin in free and purified form included within CD.

Efficient probes able to quantify antioxidant scavenging capabilities as well as ROS levels provide a key to understand antioxidants action mechanisms and to regulate the redox balance in the body (Figure 1). However, reactive species present some characteristics that make their detection difficult, like their very short lifetime and the endogenous antioxidant mechanism which regulate their levels *in vivo* [75]. Furthermore, the response of antioxidants to different radicals or oxidant sources vary widely [76]. The ideal chemical or biological probe would be highly reactive at low concentrations, specific, nontoxic, easy to use and to load into organelles, cells, or tissues without subsequent leakage, readily available and inexpensive [49, 77].

Up to now, there is not a unique probe filling all these criteria; therefore, the simultaneous use of chemical and biological methods is advised to obtain a better screening of the tested molecule properties. Chemical methods “*in tube*” provide a first approach to validate the antioxidant capacities of a specific component. On the other hand, biological methods allow measuring the antioxidant ability of a component to regulate the redox environment of an organelle and provide information about oxidative markers, intracellular antioxidant capacities, and endogenous antioxidant pathways [78].

Our results showed that the inclusion of astaxanthin within CD highly improved its lipid/aqueous affinity without affecting the intracellular antioxidant capacity against ROS. Moreover, positive antioxidant scavenging activities evidence astaxanthin cellular and mitochondrial targeted action to reduce the disturbance in the redox state of endothelial cells.

### 3.5. Indirect CD-A Antioxidant Protection against ROS

#### 3.5.1. CD-NA Protects Cells by Activation of Endogenous Antioxidant Systems by Nrf2/HO-1/NQO1 Pathway

Nrf2 (nuclear factor-erythroid 2-related factor 2) is a key transcription factor physiologically attached to Keap1 protein within the cytoplasm in basal conditions. Under oxidative stress, the Keap1-Nrf2 complex dissociates and Nrf2 translocates to the nucleus, inducing an endogenous antioxidant response of detoxifying enzymes and proteins such as heme oxygenase-1 (HO-1) and NAD(P)H:quinone oxidoreductase 1 (NQO1) [78]. Heme oxygenase-1 (HO-1) is a stress response protein induced in response to a variety of oxidative challenges and pathological stimuli having cytoprotective function. HO-1 mediates the anti-inflammatory effects [79] and has a central role in cardiovascular protection [80]. NQO1 has an anti-inflammatory action and encodes for a reductase enzyme preventing the reduction of quinones that result in the production of radical species. Inflammatory cytokines that suppress NQO1 induce oxidative stress [81, 82]; mutations in this gene have been associated with cardiovascular disorders [83].

**Figure 4:**
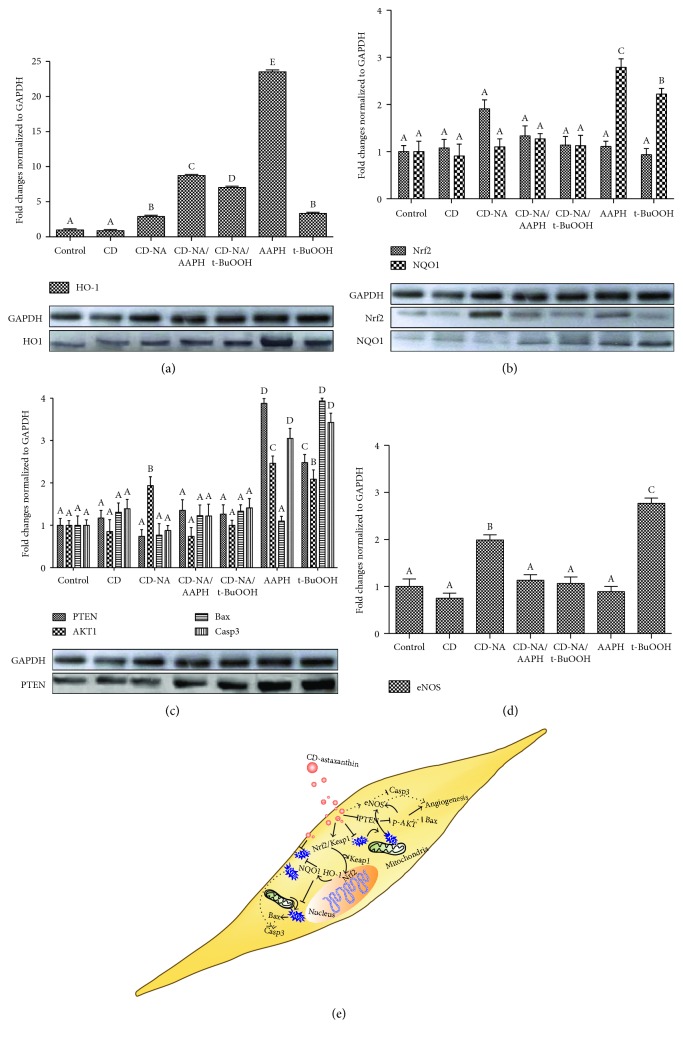
The molecular protection mechanism of CD-A complex against ROS. CD-A complex activates (a, b) Nrf2/HO-1/NQO1 and (c, d) PTEN/PI3K/AKT endogenous pathways. (e) Schematic representation of the molecular pathway of CD-A protective action on endothelial cells under oxidative stress. Levels not connected by the same letter are significantly different (*p* < 0.01).

In this research, we studied CD-NA protective effect on human endothelial cells. Two different stressors were examined: t-BuOOH and AAPH, frequently used as a free radical donors which generate a burst of ROS, thus inducing the dissociation of Nrf2/Keap1 complex [33, 84]. An overexpression of HO-1 and NQO1 was noticed when oxidative stress was induced in endothelial cells by both stressors (Figures 4(a) and 4(b)). The CD-NA cell treatments led to the upregulation of HO-1 and NQO1 basal expression and downregulation in ROS excess conditions. HO-1 and NQO1 protein expressions were analyzed after radical induction. A higher expression was detected when both stressors were present, while a similar upregulation trend using CD-NA complex was evidenced for both protein expressions (Figures 4(a) and 4(b)). In this study, the upregulation of HO-1 and NQO1 could be due to the separation of Nrf2/Keap1 by radical action, suggesting that in basal conditions, CD-NA allows Nrf2 gene upregulation. Moreover, Nrf2 gene expression was not affected after oxidative stress induction. The protein expression study confirmed these results (Figure 4(b)).

Astaxanthin action on Nrf2/OH1/NQO1 pathway was previously described *in vitro* in animal cells; however, revealing results were conflicting [22, 85, 86]. Increased cellular GSH due to astaxanthin treatment on human hepatic cellular carcinoma cells (Huh7) was not mediated by an Nrf2-dependent signal transduction pathway [87]. In contrast, Nrf2 was activated in human retinal pigment epithelial cells (ARPE-19) showing a reduction in the oxidative damage due to astaxanthin treatment [88]. Recently, Pan et al. showed that astaxanthin pretreatment significantly increased the expression of Nrf2, HO-1, and NQO1 mRNA in a cerebral ischemia rat model evidencing a protective effect against brain injuries [89]. The discrepancies between astaxanthin-reported action could be related to differences in cellular type sensitivities upon carotenoid treatment, to the type of stress inducers, and to the astaxanthin product composition [17].

#### 3.5.2. CD-NA Prevents Apoptosis of Endothelial Cells under Oxidative Stress by PTEN/PI3K/AKT Pathway

Previous studies revealed that intracellular ROS generation modulates the PTEN/PI3K/AKT pathway influencing the cell fate towards senescence and apoptosis [90]. AKT plays a vital role in vascular homeostasis, acting as a regulator of endothelial cell survival, growth, and NO production [91]. Astaxanthin was shown to protect *in vivo* isoflurane-induced neuroapoptosis in a rat model, supported by the diminution of brain damage, suppression of Casp3 activity, and upregulation the PI3K/AKT pathway [92]. In this study, ROS induction with AAPH and t-BuOOH upregulates PTEN gene expression probably to deactivate the AKT, guiding cells to apoptosis (Figure 4(c)). Conversely, CD-NA treatment significantly reduces the PTEN expression in endothelial cells under oxidative stress. Protein expression levels confirmed these results (Figure 4(c)). eNOS and Bax genes were upregulated when oxidative stress was induced using t-BuOOH stressor on endothelial cells. CD-NA treatment significantly reduced both eNOS and Bax gene expressions. This effect was not observed after AAPH ROS induction (Figures 4(c) and 4(d)). Additionally, CD-A treatment downregulates AKT and Casp3 gene expressions submitted to stress (Figure 4(c)). These results give some indications of a possible indirect CD-A cell protection against oxidative stress-induced apoptosis.

## 4. Conclusion

The use of carotenoids like antioxidants can assist the natural mechanisms of cells in neutralizing oxidative stress. Particularly, the xanthophyll carotenoid astaxanthin is of special interest, due to its ability to interact with free radicals as a chain-breaking molecule. Despite the positive results showed for astaxanthin *in vivo* treatment on induced oxidative stress-related diseases, clinical trials have been disappointing due, among other factors, to the differences between the antioxidant systems of humans and rodents. Adequate carotenoid doses and determination of appropriate length treatments are not well established yet. In addition, several inconveniences have been attributed to their prone sensibility to degradation and lack of availability. Here, we showed the capacity of CD to enhance the astaxanthin solubility, improving the astaxanthin ability to reestablish the balance in the redox state of the cell. Additionally, the direct capacity of CD-A to inhibit the HUVEC and mitochondrial ROS and to reduce lipid peroxidation was demonstrated. Moreover, the CD-A indirect action to reduce ROS levels by reinforcing the Nrf2/HO-1/NQO1 endogenous antioxidant defenses was shown. The results presented in this research were performed in a human endothelial cell line and cannot be generalized to primary cells or animal models. Owing to these results, CD-A complex appears to be highly suitable for a posterior evaluation of its antioxidants capacities to regulate the ROS production *in vivo*, as an oxidative stress regulator. However, further research is necessary before considering the possibility of using this complex in human therapies.

## Supplementary Material

Figure S1. Characterization and astaxanthin quantification into CD complex. (A) SA calibration curve acquired by UV/VIS spectroscopy showed a good linear correlation between the concentration (X) and the absorbance (Y). (B) Concentrations of astaxanthin CD complex were established by recording the absorbance in DMSO at 480 nm. (C) Weight recovery rate and (D) CD-A inclusion capacity was calculated at the end of the procedure. Trolox calibration curves were plotted to calculate (E) Trolox inhibition capacity using TEAC/ABTS assay and (F) Trolox AUC net against concentration by ORAC assay. Data are means ± SD of six experiments.

## Figures and Tables

**Table 1:** The sequences of primers used for study of profile of RNAs using q-PCR.

Target genes	Primer	Primer sequence (5′-3′)	Annealing temperature (°C)
PTEN	Forward	TCCCAGTCAGAGGCGCTATG	60
	Reverse	CACAAACTGAGGATTGCAAGTTC	

Nrf2	Forward	GAGACAGGTGAATTTCTCCCAAT	59
	Reverse	TTTGGGAATGTGGGCAAC	

HO1	Forward	ACGGCTTCAAGCTGGTGATG	61
	Reverse	TGCAGCTCTTCTGGGAAGTAG	

NQOI	Forward	ATGTATGACAAAGGACCCTTCC	62
	Reverse	TCCCTTGCAGAGAGTACATGG	

Bax	Forward	ATCCAGGATCGAGCAGGGCG	64
	Reverse	GGTTCTGATCAGTTCCGGCA	

AKT1	Forward	GTTTGCCGGAATCAATTTTC	60
	Reverse	AGCCAGAGCTGTGATCTCCTT	

GAPDH	Forward	GAGCCAAAAG GGTCATCATC	63
	Reverse	TAAGCAGTTGGTGGTGCAGG	

Caspase-3	Forward	TGTGAGGCGGTTGTGGAAGAGT	63
	Reverse	AATGGGGGAAGAGGCAGGTGCA	

eNOS	Forward	ATCTCCGCCTCGCTCATG	61
	Reverse	GAGCCATACAGGATTGTCGC	

## Additional Points

*Highlights*

1. CD improves the astaxanthin solubility without affecting its antioxidant scavenging capacities or compatibility.
2. CD-A inhibits HUVEC cellular and mitochondrial ROS by reducing the disturbance in the redox state of the cell and the infiltration of lipid peroxidation radicals.
3. CD-A complex can inhibit the oxidative stress in HUVEC by PTEN/AKT gene and protein expression and also enhance the Nrf2/HO-1 endogenous antioxidant defenses.
