# Supplementary material for: Oxidative Stress Regulation on Endothelial Cells by Hydrophilic Astaxanthin Complex: Chemical, Biological, and Molecular Antioxidant Activity Evaluation
Source: Oxid Med Cell Longev. 2017 Sep 27;2017:8073798. doi: 10.1155/2017/8073798 (PMC5635468; doi:10.1155/2017/8073798)
Supplement: Supplementary file 1 — Figure S1. Characterization and astaxanthin quantification into CD complex. (A) SA calibration curve acquired by UV/VIS spectroscopy showed a good linear correlation between the concentration (X) and the absorbance (Y). (B) Concentrations of astaxanthin CD complex were established by recording the absorbance in DMSO at 480 nm. (C) Weight recovery rate and (D) CD-A inclusion capacity was calculated at the end of the procedure. Trolox calibration curves were plotted to calculate (E) Trolox inhibition capacity using TEAC/ABTS assay and (F) Trolox AUC net against concentration by ORAC assay. Data are means ± SD of six experiments. [file 8073798.f1.pdf]

Supporting information

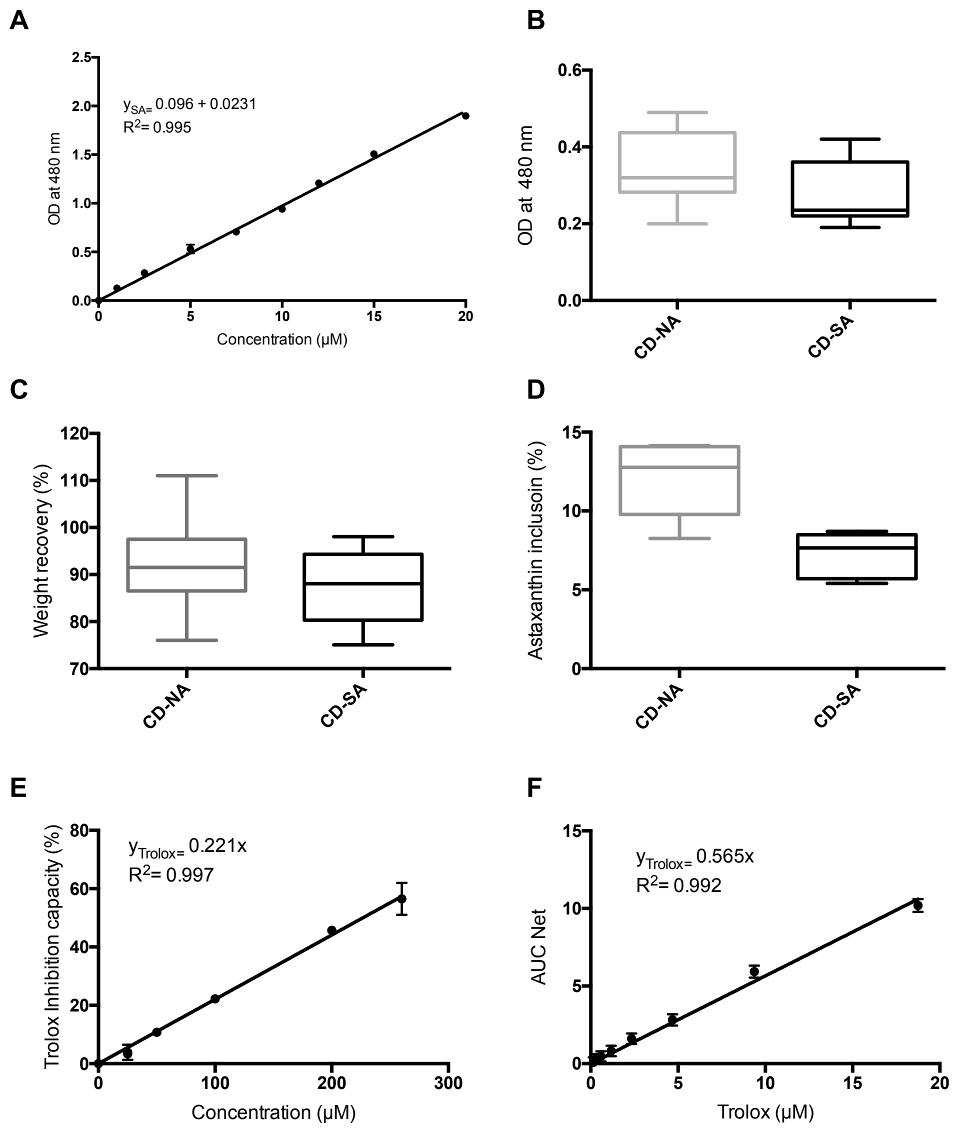

Figure S1. Characterization and astaxanthin quantification into CD complex. (A) SA calibration curve acquired by UV/VIS spectroscopy showed a good linear correlation between the concentration (X) and the absorbance (Y). (B) Concentrations of astaxanthin CD complex were established by recording the absorbance in DMSO at 480 nm. (C) Weight recovery rate and (D) CD-A inclusion capacity was calculated at the end of the procedure. Trolox calibration curves were plotted to calculate (E) Trolox inhibition capacity using TEAC/ABTS assay and (F) Trolox AUC net against concentration by ORAC assay. Data are means ± SD of six experiments.
